# Forest biomass accumulation is an important source of acidity to forest soils: Data from Swedish inventories of forests and soils 1955 to 2010

**DOI:** 10.1007/s13280-021-01540-y

**Published:** 2021-03-29

**Authors:** Erik Karltun, Johan Stendahl, Johan Iwald, Stefan Löfgren

**Affiliations:** 1 Department of Soil and Environment, SLU, Box 7014, 750 07 Uppsala, Sweden; 2 Department of Aquatic Sciences and Assessment, SLU, Box 7050, 750 07 Uppsala, Sweden

**Keywords:** Atmospheric deposition, Biological acidification, Critical loads, Forest harvest, Standing biomass, Soil impact

## Abstract

The input of acidity to Swedish forest soils through forestry between 1955 and 2010 is compared with the acid input from atmospheric deposition. Depending on region, input of acidity from forestry was the minor part (25–45%) of the study period’s accumulated acid input but is now the dominating source (140–270 mol_c_ ha^−1^ year^−1^). The net uptake of cations due to the increase in standing forest biomass, ranged between 35 and 45% of the forestry related input of acidity while whole-tree harvesting, introduced in the late 1990s, contributed only marginally (< 2%). The geographical gradient in acid input is reflected in the proportion of acidified soils in Sweden but edaphic properties contribute to variations in acidification sensitivity. It is important to consider the acid input due to increases in standing forest biomass in acidification assessments since it is long-term and quantitatively important.

**Supplementary Information:**

The online version contains supplementary material available at 10.1007/s13280-021-01540-y.

## Introduction

The atmospheric deposition of sulfuric acid, measured as the non-sea salt sulfate (SO_4_^2−^) deposition, was the major source of anthropogenic ecosystem acidification during the last century. Due to the successful implementation of air pollution emission controls, the atmospheric deposition of SO_4_^2−^ has decreased tremendously since it peaked in the late 1970s. In Europe, it has reduced by about 70% compared even with the levels in the beginning of the twentieth century (Engardt et al. 2017). In Sweden, the wet deposition of non-sea salt SO_4_^2−^ was approximately 2.2 times higher at the peak in the early 1970s compared with 1955, the first year of extensive collection of deposition data. Currently, the wet deposition of SO_4_^2−^ in Sweden is around 13% of the peak value, corresponding to less than 2 kg S ha^−1^ year^−1^ (Ferm et al. 2019). Following the reduced deposition of acidity since the early 1990s, pH and acid neutralizing capacity (ANC) in soil solution (Pihl Karlsson et al. 2011; Akselsson et al. 2013; Johnson et al. 2018) and surface waters (Futter et al. 2014; Vuorenmaa et al. 2018) are recovering from acidification throughout Europe. Similar pH and ANC recovery trends are documented in surface waters in North America (Garmo et al. 2014; Driscoll et al. 2016).

Following the strong decline in acid deposition there are few observations of forest soil recovery from acidification despite the recovery trends in soil solution and fresh waters. Instead, the results are inconclusive and the trend direction varies depending on chemical variable and soil horizon. This was exemplified by the study of Lawrence et al. (2015) who evaluated soil data from 27 forest sites in eastern Canada and northeast US, experiencing wet SO_4_^2−^ deposition reductions in the range 6–76% over intervals of 8–24 years. They showed a statistically significant (*p* < 0.1) positive correlation between soil pH and reduction in SO_4_^2−^ deposition only in the B horizon and when the deposition reduction was greater than 40%. Base saturation, exhibited a positive correlation (*p* < 0.01) with SO_4_^2−^ deposition reduction only in the O horizon and a negative correlation in the B horizon. Exchangeable calcium (Ca^2+^) showed no relation with SO_4_^2−^ deposition in any soil horizon. (Lawrence et al. 2015).

The results from the Swedish Forest Soil Inventory (SFSI) have also been inconclusive. The difference in base saturation in the O and B horizons between the first two inventories performed 1983–87 and 1993–98, respectively, were small. However, other variables such as pH_H2O_ and exchangeable Al^3+^ were both increasing which is contradictory (Karltun et al. 2003). A later assessment, including results from the inventory performed 1999–2003, showed a statistically significant pH_H2O_ increase of approximately 0.1 units per decade for the B horizon except for the north and the southwest parts of the country attributed to low and high atmospheric acid deposition, respectively. These pH trends were supported by decreased exchangeable acidity, while the trends for exchangeable base cations, Al^3+^ and base saturation were non-significant in the B-horizon (Stendahl 2007).

The lack of a consistent trend for various soil chemical variables and horizons, is expected since other processes besides atmospheric deposition influence soil acidity and nutrient content. A process of considerable quantitative importance is biological acidification (Nilsson 1993). Biological acidification occur when plants takes up more cations than anions (net cation uptake). This uptake results in a corresponding release of H^+^ from the plant to the soil in order to maintain charge balance (Nilsson et al. 1982; van Breemen et al. 1984).

Since the 1950s, the total annual volume increment, including harvest, has increased from approximately 0.8 × 10^8^ to 1.2 × 10^8^ m^3^ in the productive forests of Sweden, i.e. forests with a potential stem wood growth of > 1 m^3^ ha^−1^ year^−1^ (SLU 2018). Hence, the acidification pressure from tree growth has persisted while the SO_4_^2−^ deposition reduced significantly. Iwald et al. (2013) showed that the acidifying effect of harvesting Scots pine (*Pinus sylvestris*, L.) and Norway spruce, (*Picea abies*, (L.) H. Karst) corresponds to 57–108% and 114–263%, respectively, of the maximum acid deposition during the period 1996–2009 using data from the Swedish National Forest Inventory (Claesson 2008).

In studies on the acidifying effect of forestry the focus is on acidification through export of harvested products and whole-tree harvesting (Akselsson et al. 2007; Aherne et al. 2012). The Simple Mass Balance (SMB) critical load (CL) equation originally includes vegetation uptake of base cations and nitrogen (CLRTAP 2017), but for several CL estimates related to harvest intensity only the BC and N uptake removed by harvest is considered disregarding potential changes in the standing biomass (Moldan et al. 2017; Akselsson and Belyazid 2018). There are some important aspects to consider here. Firstly, the transfer of acidity from the biomass growth to the soil does not occur at harvest but when the trees grow, i.e. it has already taken place at the time of harvest. Secondly, it is the growth rate that determines the acid input to soil, not the harvest rate. If harvest rates were constant over time and roughly equal to growth rate one could ignore these factors. However, tree growth continues to exceed the harvest rate and harvest rates also vary considerably with market fluctuations and natural disturbances.

In Sweden, the forest volume has increased from 2.2 × 10^9^ m^3^ in 1955 to 3.0 × 10^9^ m^3^ in 2010. During this period, the annual gross felling was less than the gross increment even though the felling increased from 50 to 90 × 10^6^ m^3^ year^−1^ (Swedish Forest Agency 2014). In Europe, standing volume exhibits a similar trend and the growing stock increased by 7 × 10^9^ m^3^ between 1950 and 2000 in Western Europe and with 2 × 10^9^ m^3^ in Eastern Europe excluding the Baltic States and four of the five countries of the former Yugoslavia. During this period the biomass harvest decreased from c. 90 to 80% of the annual increment in Western Europe and from 80 to 70% in Eastern Europe (FAO 2005). In the US, the standing volume in forests increased as well, from 17.4 to 23.6 × 10^9^ m^3^ between 1953 and 1997. Similarly to Europe, the annual biomass harvest did not exceed the annual increment during this period. Actually, the annual biomass harvest increased from 0.34 × 10^9^ in 1953 to 0.45 10^9^ m^3^ year^−1^ in 1997 corresponding to 85% and 68% of the annual increment, respectively (USDA 2001).

The aim of this paper is to quantify the acid input to forest soils through biological acidification by forestry between 1955 and 2010 and to compare that input with the acid load through atmospheric deposition. We separate the biological acidification in two categories; (i) input of acidity from net accumulation of cations in standing biomass and (ii) input of acidity through biomass harvest of stems and harvest residues. Finally, we assess the balance between the acid input from deposition and forest growth, the stock of base cations in biomass and the exchangeable base cation storage in soils, and discuss how this balance may be influenced by edaphic factors.

## Materials and methods

The information in this section is a summary of the data materials used for the calculations. A full account of the data material and methods is found in the Supplement.

### Forest biomass, harvesting and harvest residues data

Data from the Swedish NFI for the period 1955–2010 were used to quantify biomass stocks and harvests of Scots pine, Norway spruce and birch (sum of *Betula pendula*, Roth and *Betula pubescens*, Ehrhart), which together make up 95% of the timber volume in Sweden (SLU 2017). Harvest residues extraction was calculated for the period 1999–2010 based on statistics of the clear-cut area where harvest residues were removed for the entire period 1999–2010 and combined with data on extracted volumes from 2007 to 2010 (Swedish Forest Agency 2014).

Changes in the net cation and anion stocks in standing biomass and export through harvest were calculated based on data on the concentration of base cations (Ca^2+^, Mg^2+^, K^+^, Na^+^) and anions (H_2_PO_4_^−^, SO_4_^2−^, Cl^−^) in the different tree fractions compiled from different studies in Sweden and Finland using the data described in Iwald et al. (2013). The net cation uptake is defined as the amount of base cation mole charge equivalents minus the amount of anion mole charge equivalents taken up in the tree biomass.

### Soil variables

The stock of exchangeable base cations in the humus layer and in the mineral soil down to 50 cm depth (BC soil), for mass-balances often assumed as root depth, was calculated using data from 3618 plots from the SFSI collected during the period 2003–2012. Based on this data, the proportion of acidified soils was calculated at county level. We have used the definition of acidified soil suggested by the Swedish Forest Agency (Gustafsson et al. 2001). The criteria is that ${\text{pH}}_{{{\text{H}}_{2} {\text{O}}}} < 4.5$ in the B-horizon or a ${\text{pH}}_{{{\text{H}}_{2} {\text{O}}}} < 4.75$ in the C-horizon and it is based on the argument that there are no natural soil forming processes or parent material properties that could result in so low pH levels in well-drained Swedish forest soils.

### Deposition of acidity

The acidity in deposition (mol_c_ ha^−1^) was estimated from the deposition of non-sea salt SO_4_^2−^ and non-sea salt Ca^2+^ according to:

$${\text{acidity}} = {\text{SO}}_{4}^{{2 - }} - {\text{Ca}}^{{2 + }}$$

We use the following assumptions:

- The origin of non-sea salt SO_4_^2−^ is 100% sulphuric acid (H_2_SO_4_) formed in the atmosphere.
- Non-sea salt Ca^2+^ is derived from Ca^2+^ containing substances with a liming effect, e.g. CaO, corresponding to two hydroxide ion (OH^−^) equivalents per Ca^2+^.
- The acidification effect by N deposition is negligible due to approximately equal deposition of NH_4_^+^ and NO_3_^−^ (see Supplement)
- Leaching of NO_3_^−^ is negligible in Sweden (see Supplement)
- Other cations and Cl^−^ are assumed acidity neutral and to originate from sea salt (Ferm et al. 2019).

### Statistics

The plot based sampling design of the NFI was the basis of the estimations (Fridman et al. 2014). This sampling design was introduced in the NFI in 1953 and has remained relatively unchanged since then, with the addition of permanent plots in 1983 where the SFSI is carried out. During the study period 1955–2010, the inventory has covered the entire country annually with plots that are organized into sample plot clusters (i.e. tracts), utilizing a stratified sampling intensity with less intense sampling towards the north according to 5 regionalized strata. Moving five-year averages were used for the time-series of the forest data. Linear regression was used to test trends in acid input and the effect of acid input on soil acidification status.

## Results

**Fig. 1:**
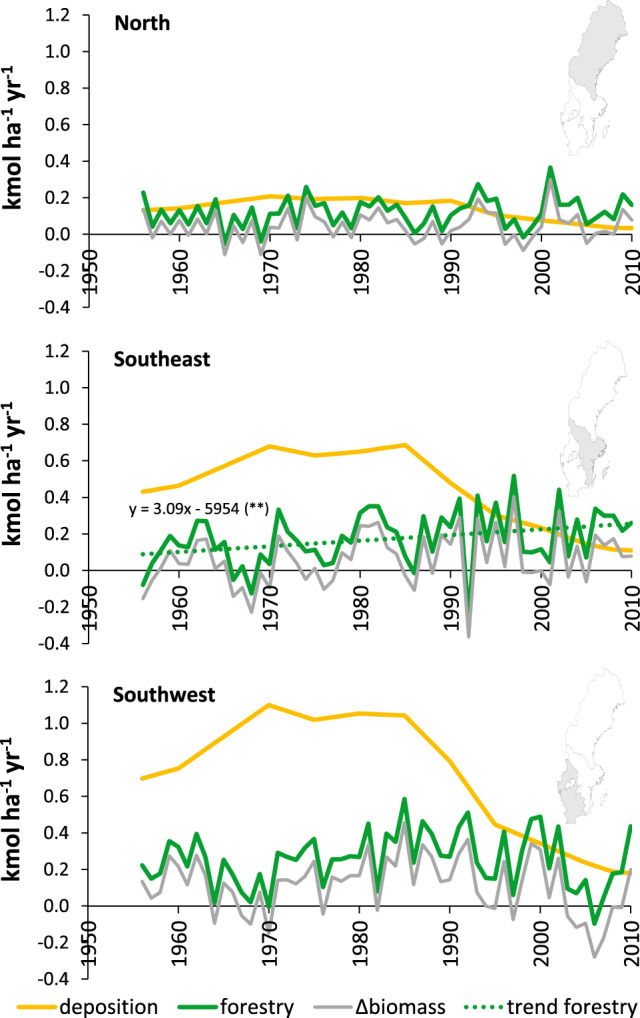
Acid input to forest land through forestry and atmospheric deposition. The effect of forestry is shown as total effect (net cation removal in standing biomass plus harvest including harvest residues for bioenergy) and as an effect of change in standing biomass only. The regression line is the trend in total forestry related input (biomass uptake and export). Only significant (*p* < 0.05) trends are shown

The deposition of acidity increased considerably during the 1950s and 60s and reached peak levels in the 1970s and 80s, after which it decreased successively to low levels in 2010 (Fig. 1). Throughout the studied period, southwest Sweden received the largest deposition of acidity. During the peak period the amount was 1100 mol_c_ ha^−1^ year^−1^, while this figure was 680 and 210 mol_c_ ha^−1^ year^−1^ in southeast and north Sweden, respectively. In 2010, the deposition of acidity in these regions had decreased to 180, 110 and 30 mol_c_ ha^−1^ year^−1^, respectively.

**Fig. 2:**
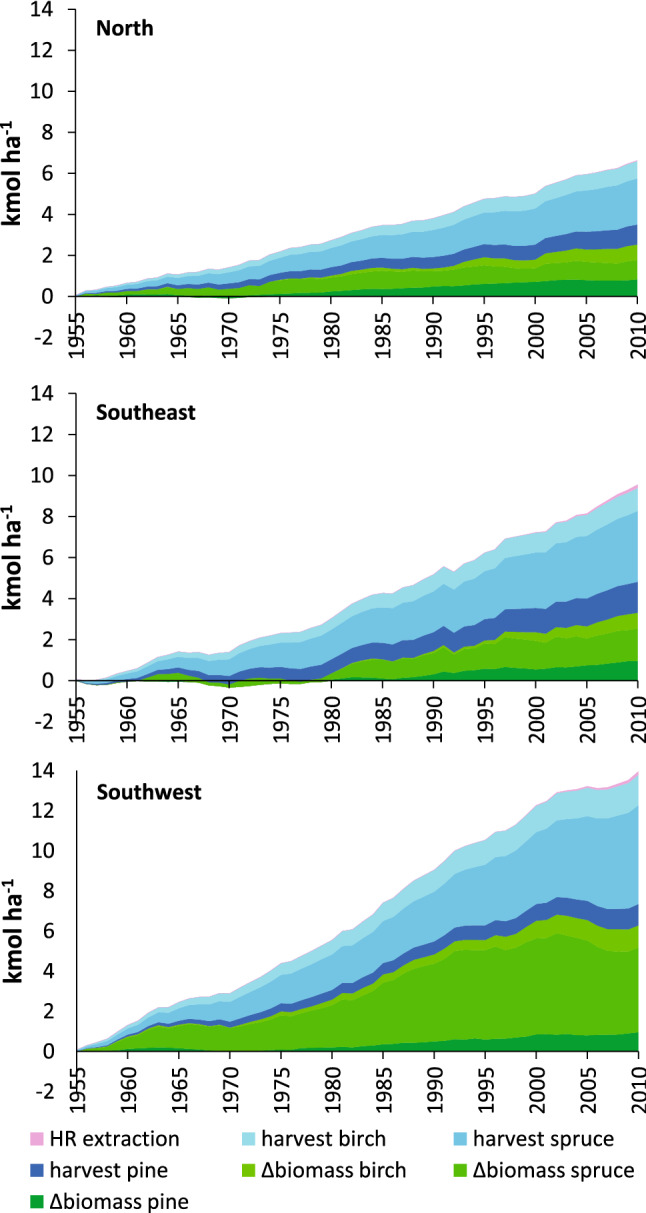
Accumulated acid load to soil due to net cation removal due to changes in standing biomass and harvest between 1955 and 2010 for Scots pine, Norway spruce and birch and the extraction of harvest residues (HR) for bioenergy purposes

In contrast to atmospheric deposition, the acid input related to harvest (removed biomass) and accumulation of standing biomass remained at the same level throughout the study period except for the southeast of Sweden where an annual increase of 3.6% per year was statistically significant (*p* < 0.01, Fig. 1). In this region, tree growth and harvest rate were roughly equal during the first 25 years of the time series for Norway spruce, resulting in less increase in standing biomass and an acid input to the soil proportional to the amount of harvested wood (Fig. 2). Thereafter, the standing biomass increased, adding acidity to the soils in amounts corresponding to the export of net cation uptake in harvested biomass. The acid input from forestry increased from 90 to 260 mol_c_ ha^−1^ year^−1^ in southeast (Fig. 1). In the other two regions, the net biomass increased throughout the period, adding acidity to the soils at similar rates as harvest. In the southwest and northern regions, the acidity load from these forestry sources was on average 250 and 120 mol_c_ ha^−1^ year^−1^, respectively. Depending on region, input of acidity from forestry was the minor part (25–45%) of the study period’s accumulated acid input (Fig. 4) but is now the dominating source (140–270 mol_c_ ha^−1^ year^−1^, Fig. 1).

There was a strong inter-annual variation in tree harvest levels (Fig. 1), which occasionally caused substantial reductions in the standing biomass. This was most evident for the southwest region, where a severe storm occurred in January 2005.

Among the tree species, the biomass increase and harvest of Norway spruce was the most important source of acid input to soils in southwest Sweden (Fig. 2). In the other two regions, the acid input from Scots pine, Norway spruce and birch was more evenly distributed. Besides being the most common tree species in southern Sweden, different tree fractions of Norway spruce contain more BC than the corresponding fractions of Scots pine (Table 1). Birch is the most acidifying tree species in per unit of biomass due to high concentrations of BC in different tree fractions (Table 1). However, the standing biomass of birch is lower than for Norway spruce and Scots pine, keeping the amounts of acidity added from birch to soils at lower levels in southwest Sweden. In the other two regions, the large net BC uptake during growth compensates for this and make the acid input by birch comparable to that of Scots pine and in the northern region also with Norway spruce (Fig. 2).

**Table 1:** Net cation uptake per m^3^ produced wood for Scots pine, Norway spruce and birch

	Scots pine	Norway spruce	Birch
	mol_c_ m^−3^		
Stem	22	36	74
Top and branches	31	84	40
Stump	6	9	22

**Fig. 3:**
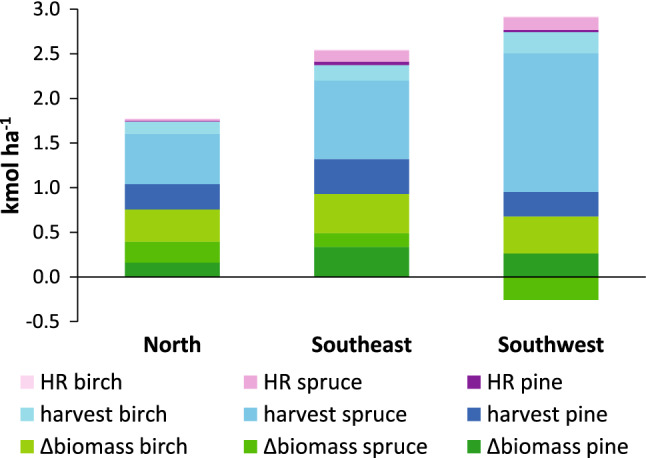
Accumulated acid input (net cation removal) on forest land as an effect of change in standing biomass and harvest between 1999 and 2010 for Scots pine, Norway spruce and birch including the extraction of harvest residues (HR) for bioenergy purposes. The negative values on Norway spruce biomass in the southwest region is due to the extensive harvesting after the storm-felling in 2005

The removal of harvest residues for bioenergy purposes was taken into account for 1999 and onwards. Before that, the extraction of harvest residues was low. Compared with the accumulated acid input since 1955 related to stem-only harvest and increase in tree biomass, the accumulated acidity related to extraction of harvest residues is low in the two southern regions (< 2%), and negligible in the northern region (< 1%, Fig. 2). However, during the period 1999–2010, when whole-tree harvesting became more common, the extraction of harvest residues became a noticeable but still low contributor to soil acidification (Fig. 3).

**Fig. 4:**
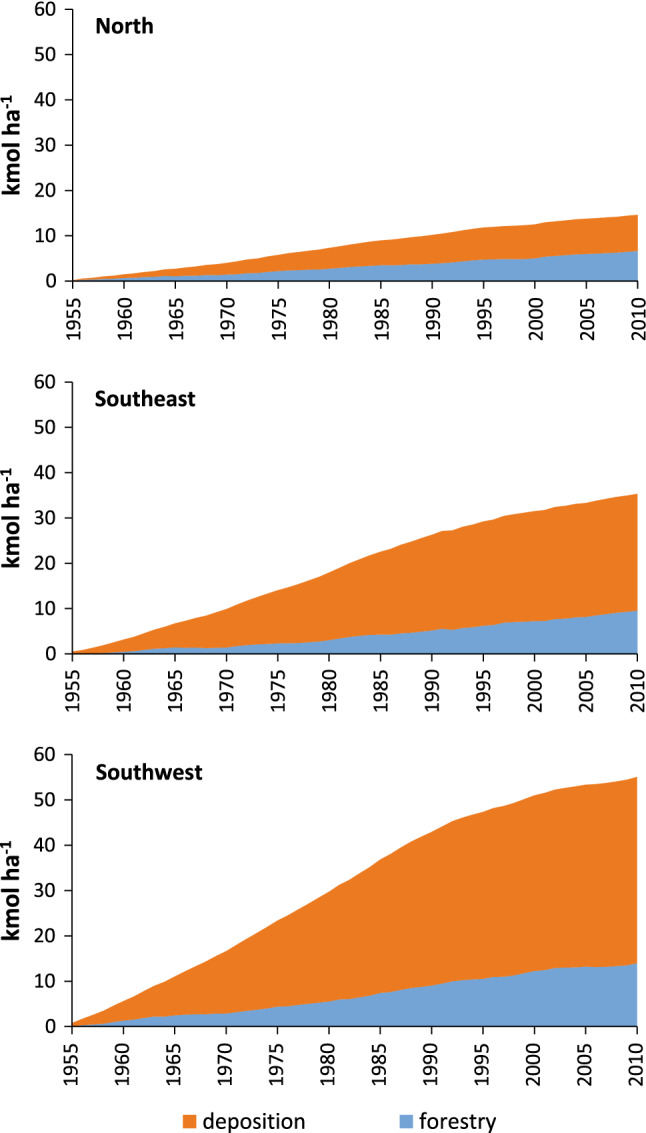
Accumulated acid input on forest land as an effect of acid deposition and forestry (net cation removal due to change in standing biomass and harvest) between 1955 and 2010

From the estimates above, it is evident that the acid input to soils from forestry currently exceeds those from atmospheric deposition in productive forests over the entire Sweden. However, the accumulated acidity load from acid deposition since 1955 is much larger than the acidity load from forestry in the two southern regions, while the two sources are of similar magnitude in the northern region (Fig. 4). The accumulated acidity load from both atmospheric deposition and forestry decreased from southwest to the north of Sweden. Along this gradient, the sum of these sources decreased from 55 kmol_c_ ha^−1^ in southwest, 35 kmol_c_ ha^−1^ in southeast to 15 kmol_c_ ha^−1^ in the north (Fig. 4). The share of accumulated acidity from forestry between 1955 and 2010 were 25% for the southwest region, 27% for the southeast region and 45% for the north region.

**Fig. 5:**
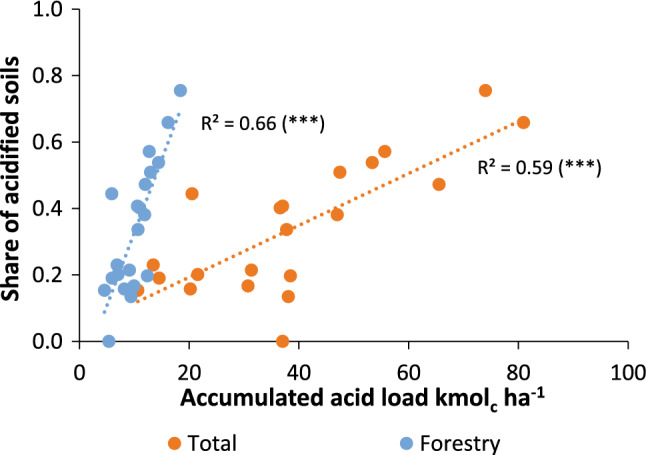
Proportion of acidified forest soils as a function of accumulated acid input 1955 to 2008 from forestry (blue) and total acid input (orange)

The share of forest soils classified as *acidified* at county level, has statistically significant (*p* < 0.001) linear relations with both the total acidity load (*r*^2^ = 0.59) and with the contribution from forestry alone (*r*^2^ = 0.66, Fig. 5). Generally, the acid input and the share of acidified soils decreased from south to north. However, counties with a high proportion of soils with parent material influenced by CaCO_3_ or a large proportion of post-glacial and glacial clays deviate from this picture with none or a smaller proportion of acidified soils.

**Fig. 6:**
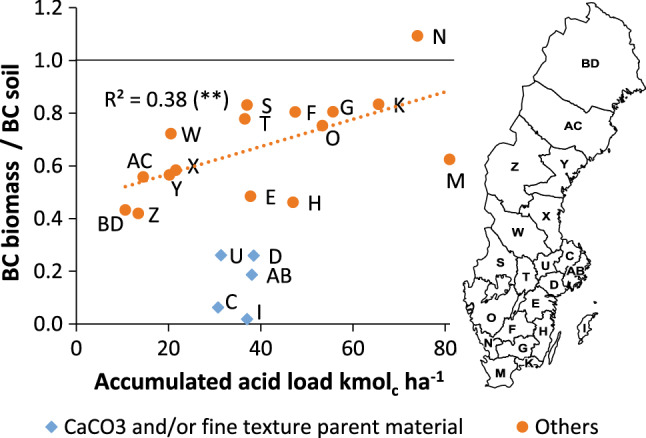
The ratio between the base cation pool in the tree biomass and in the soil (salt extractable) as a function of total accumulated acid input 1955 to 2008. Counties with influence of CaCO_3_ or high clay content are presented as a separate group

The intensity of soil acidification in relation to forest biomass base cation accumulation can be expressed using the ratio of the amount of base cations in forest biomass and the exchangeable pool of base cations in soils (BC_biomass_/BC_soil_). Looking at data in Fig. 6 from the 21 counties in Sweden there is no clear relationship between the total amount of acid input and the BC_biomass_/BC_soil_ ratio. For counties that historically have experienced a strong acid deposition load (K, M, N, O, F, G) the soils have been depleted and a higher relative share of the base cation pool is found in the biomass. For the county with the single highest acidity load, Halland (N), more base cations were accumulated in the tree biomass than in the exchangeable soil pool (ratio > 1, Fig. 6).The five counties with a legacy influence of CaCO_3_ in till soils or having a high clay content (AB, C, D, I, U) (Figure S1 and S2), have low BC_biomass_/BC_soil_ ratios (Fig. 6). If these counties are exempted in a linear regression analysis there is a statistically significant relationship (*r*^2^ = 0.38, *p* < 0.01) between the total acid input and the BC_biomass_/BC_soil_ ratio.

## Discussion

Here we make a complete assessment of the total acidity load from forestry including not only the effects of biomass harvest but also the acidifying effect of the gradually increasing standing volume. A similar approach was taken by Nilsson (1993) but availability of more detailed and more recent data merits a new analysis.

The standing volume in both Europe and the US has increased at the same time as the export of biomass from the forests has increased (USDA 2001; FAO 2005), which means that biological acidification has increased over time. Total acidity load to soils from forestry is currently exceeding acid deposition (Fig. 1), while the opposite holds true for the accumulated acidity load 1955–2010 (Fig. 4). Since the NFI was initiated in 1922, the annual cuttings and the standing volume has successively increased (Swedish Forest Agency 2014), indicating a forestry induced acidity load spanning a period that is longer than the peak in acid deposition caused by the use of fossil fuels during the second half of the twentieth century.

The critical load (CL) concept, adopted by the UN Convention on Long-Range Transboundary Air Pollution (CLRTAP), includes in the mass-balance atmospheric deposition of acidifying sulfur and nitrogen compounds and the acidity load from net uptake of base cations in biomass defined as “the net uptake by vegetation that is needed for long-term average growth” (CLRTAP 2017). Net uptake is the gross uptake minus the uptake required to replace losses through litterfall and root exudation and would include accumulation in an increasing amount of biomass. The uptake and leaching are balanced by input from weathering and deposition of base cations. However, in applications of CL aiming at evaluating long-term sustainability of forestry the base cation uptake term is equated with the export of base cations through harvest (Aherne et al. 2012; Moldan et al. 2017). A recent study also suggested a new CL concept, “Critical biomass harvesting”, that does not account for the accumulation of base cations in biomass but include base cation export through harvest (Akselsson and Belyazid 2018). Common to the different CL calculations is that they ignore the anion uptake, which is an alkalizing effect that we have included in our study.

The CL concept handles the input and output fluxes of BC, but the available BC reserves in soils are overlooked (CLRTAP 2017; Akselsson and Belyazid 2018). Hence, at a negative BC balance, we do not know whether CL exceedance may occur within 1, 10 or 100 forest rotations (Rosenstock et al. 2019) or if non-salt extractable BC soil sources are available (Bel et al. 2020), which is essential for assessing the severity of the CL exceedance and necessary to consider for advice on forest management. The non-existing relation between total acidity load and BC pools for those counties with CaCO_3_ influenced or fine textured soils highlights this shortcoming (Fig. 6).

The increased demand for bioenergy has put focus on whole-tree harvest and the net withdrawal of base cations and acidity load from extraction of branches and tops compared with conventional stem-only harvest (Thiffault et al. 2011; Iwald et al. 2013; Achat et al. 2015; Zetterberg et al. 2016). The results from this study show that at regional level, the acidity load from bioenergy harvest is small compared with the acidity load from increased standing volume and stem-only harvest in the temporal perspectives of 55 years (Fig. 2) or a decade (Fig. 3). The reason is that bioenergy fractions are only harvested at a restricted area of the harvested sites. At stand level, however, where biomass extraction for bioenergy constitutes a large fraction (c. 30%) of the net base cation export, the acidity load is quantitatively important (Iwald et al. 2013; de Jong et al. 2017).

The mobility of acidity down through the soil profile is controlled by the availability of mobile anions (Iwald et al. 2013). Without accompanying anions, the acid input related to forest growth is mainly affecting the upper soil horizons. Elevated SO_4_^2−^ deposition results in transport of acidity down through the soil profile (van Breemen et al. 1983; Löfgren et al. 2017). However, high chloride (Cl) deposition, often episodic in connection with storm events, also contributes with mobile anions (Hindar 2005; Akselsson et al. 2013), especially on the Swedish west coast and its hinterland (Nilsson 1993). This Cl deposition facilitates transport of biologically generated acidity to deeper soil horizons.

Paleolimnological records have shown that land-use changes have an impact on the alkalinity status of lakes with periods of deforestation and cultivation associated with higher pH in lakes (Renberg et al. 1993). It is likely that the ongoing increase in standing biomass is having an impact on stream and lake water chemistry that may have ecological impacts on freshwater biota, especially when compared to the pre-industrial period characterized by an intensive expansion of agriculture and deforestation resulting in higher pH. However, nothing suggests that the effects of forestry would have the detrimental impacts on freshwaters that the acid deposition had in the end of the twentieth century.

The data from the Swedish forest soil survey 2003–2012 exhibited a clear southwest-north gradient in acid–base status (Fig. 5), which is similar to the spatial pattern found in a study of deep soil profiles by Karltun (1994) and an evaluation of data from the Swedish forest soil survey 1993–2002 (Karltun et al. 2003). However, the geographical covariation between acid deposition and forest growth makes it difficult to separate the influence of those two acidity sources on soil status in the upper part of the soil profile.

The difficulty to separate the two sources is demonstrated by the similar positive relationships between the proportion of acidified forest soils and the accumulated acidity load from forestry and the accumulated total acidity load also including acid deposition (Fig. 5). However, it is important to remember that looking at the entire study period, the relative contribution of acid deposition, 75% and 73%, has come from acid deposition in the southwestern and southeastern regions, respectively as indicated by the large difference in slope between the two regression lines. The input of acidity from forestry has been quite stable over time while the deposited acidity first increased and thereafter decreased during the study period. Hence, the reason that forestry input 2010 is higher than the deposited acidity is more due to the fact that the deposited acidity has decreased, and less due to increase in standing biomass and increased export of harvested products.

The ratio between current pool of base cations in tree biomass and the exchangeable base cation pool in the soil increased linearly with accumulated acidity load for counties with few soils with CaCO_3_ influence or high clay content in the parent material. This is in agreement with data from experimental sites in Europe and North America, which show an increased base cation fraction with tree age (≈ standing volume), ultimately exceeding the exchangeable base cation pools in the soil (Watmough et al. 2005; Richardson et al. 2017). The county of Halland (N) is the only county that has more base cations in the trees than in the soils (BC_biomass_/BC_soil_ ratio > 1). Halland has had the highest historical acid deposition and has the largest standing volume of all counties in Sweden, 190 m^3^ ha^−1^ as compared with the average 143 m^3^ ha^−1^. Additionally, the share of Norway spruce with high base cation concentrations in tree fractions is larger there than in the rest of the country, 52% and 41%, respectively (Swedish Forest Agency 2014). Further, counties with forest soils with parent material dominated with a poorer mineralogy (glacial till or glacifluvial sediments derived from gneiss and granite bedrock) and an acid input exceeding 35 kmol_c_ ha^−1^ (Fig. 6) generally have BC_biomass_/BC_soil_ ratios above 75%, while the northern counties (AC, BD, Z and W), despite poor mineralogy and low cation exchange capacity due to lower amounts of soil organic matter, have lower ratios.

## Conclusions

Based on Swedish data on forest growth and atmospheric deposition of acidity for the period 1955–2010, we show that the acidity load from forest growth has been a significant and rather constant source of acid input to forest soils throughout the period. It was higher than the contribution from acid deposition at the end of the study period. Atmospheric deposition dominated the accumulated acidity input for the study period in the southwest and southeast regions of Sweden, but not in the north. The forestry induced acidity load is almost evenly originating from stem wood harvest and increased standing volume, while the increased use of harvest residues for bioenergy purposes is a less important source for the acidity load at regional level. It is, however, of significance at stand level. We argue that the acid input to the soil from the substantial increase of standing forest biomass is important to include in acidification assessments since it is long-term and quantitatively important. An international outlook indicates that increased standing volume could be important also for the acidity load to forest soils in many European countries and the US. Critical load estimates and mass-balances at regional or national levels are performed without taking into account the effects of long-term changes in standing volumes and BC reserves in the soils. Hence, a reevaluation of the forestry impact on the CL concept seems reasonable. In Sweden, soil mineralogy and the total input of acidity can explain much of the variation in the distribution of base cations between tree biomass and soil.

## Electronic supplementary material

Below is the link to the electronic supplementary material.

Electronic supplementary material 1 (PDF 562 kb)
