# Supplementary material for: Forest biomass accumulation is an important source of acidity to forest soils: Data from Swedish inventories of forests and soils 1955 to 2010
Source: Ambio. 2021 Mar 29;51(1):199–208. doi: 10.1007/s13280-021-01540-y (PMC8651924; doi:10.1007/s13280-021-01540-y)
Supplement: Supplementary file 1 — Electronic supplementary material 1 (PDF 562 kb) [file 13280_2021_1540_MOESM1_ESM.pdf]

Ambio Electronic Supplementary Material

This supplementary material has not been peer reviewed.

Title: **Forest biomass accumulation is an important source of acidity to forest soils – data from Swedish inventories of forests and soils 1955 to 2010**.

Authors: Erik Karltun, Johan Stendahl, Johan Iwald and Stefan Löfgren

Material and methods

Forest biomass, harvesting and harvest residues data

Data from the Swedish National Forest Inventory (NFI) for the period 1955-2010 were used to quantify biomass stocks and harvests of Scots pine (*Pinus sylvestris*, L.), Norway spruce *Picea abies*, ((L.) H. Karst) and birch (sum of *Betula pendula*, Roth and *Betula pubescens*, Ehrhart), which together make up 95% of the timber volume in Sweden (SLU 2017). The calculations were carried out at county level and the results were then aggregated into three regions: southwest, southeast and north (main article, Figure 1). Annual changes in biomass stocks were calculated for different tree fractions; stem wood, bark, branches, needles, roots and stumps. The total biomass for the different tree compartments was calculated based on biomass expansion functions (Marklund 1988; Petersson and Ståhl 2006). Statistics from the Swedish NFI on annual stem harvest of Scots pine, Norway spruce and birch were available for the three regions for the period 1955-2010 (SLU 2017). Within each of the three regions it was assumed that the harvests at county level was distributed in proportion to the timber storage, accounting for tree species.

Harvest residues extraction was calculated for the period 1999-2010 based on statistics of the clear-cut area where harvest residues were removed for the entire period 1999-2010. These data were combined with statistics on extracted volumes that was available only for 2007-2010 as three-year moving averages (Swedish Forest Agency 2014). Hence, the ratio between extracted volume and area from 2007-2010 was used to estimate the extracted volumes during 1999-2006, i.e. for the period when only area statistics were available. Furthermore, area statistics was available only at the regional level, while volume statistics was available at the county level. Thus, the extraction of harvest residues was allocated to the different counties in proportion to each county's share of the extracted volume in the region during 2007-2010. The calculations were made separately for Scots pine, Norway spruce and birch under the assumption that the proportions between the tree species were the same for the extraction of harvest residues as for harvested stem wood. The conversion from volume to biomass was made according to the relationship 1 m^3 of harvest residues = 0.17 ton DM biomass (Swedish Forest Agency 2014). The distribution of the harvest residues between branches and needles was estimated according to biomass expansion functions (Marklund, 1988). Consequently, pine was assumed to have a proportion of 86% branches and 14% needles while spruce was assumed to have a proportion of 75% branches and 25% needles. Harvest residues of birch were assumed to consist only of branches, no leaves.

The forestry induced acidity load is determined by the accumulated net cation uptake during stand development. The principal difference between the acidity input created by increased standing volume and the one balanced by biomass harvest and export is that the latter induces a permanent loss of ANC corresponding to the amounts of base forming elements in the biomass export. In non-dynamic mass-balances such as CL models (Akselsson and Belyazid 2018) this loss of ANC, equivalent to the soil acidity load, used to be accounted for the year when the harvest occurred. In this work, we have used the same method even though the acidity load was built up during forest growth, starting long before harvest. Due to inter-annual variations in felling, this simplification induces net variation between years in acidity load since biomass harvest varies more between years than growth do.

Changes in the net cation and anion stocks in standing biomass were calculated based on data on the concentration of base cations (Ca^{2+}, Mg^{2+}, K^+, Na^+) and anions ($H_2PO_4^-$, SO_4^{2-}, Cl^-) in the different tree fractions compiled from different studies in Sweden and Finland using the data described in Iwald et al. (Iwald et al. 2013). The net cation uptake (ΔBC) is defined as the amount of base cation charge equivalents minus the amount of anion charge equivalents taken up in the tree biomass. It was calculated by subtracting the anions according to the following:

$$\Delta BC = (Ca^{2+} + Mg^{2+} + K^+ + Na^+) - (H_2PO_4^- + SO_4^{2-} + Cl^-) \qquad [charge\ equivalents]$$

Deposition of N occur as ammonium (NH_4^+), with an acidifying effect through tree uptake, and nitrate (NO_3^-), with an alkalizing effect through tree uptake. Since there is insignificant NO_3^- leaching in Swedish forests, even in the southwest of Sweden where N deposition is quite high (Futter et al. 2010; Löfgren et al. 2014; Högberg et al. 2017) and since available deposition data for NH_4^+ and NO_3^- show that the two ions are deposited in approximately equal proportions (Ferm et al. 2019), deposition and uptake of N was assumed not to cause any net acidification. Based on harvested biomass of Scots pine, Norway spruce and birch for each county, the net cation export due to stem harvesting was calculated using the concentrations of cations and anions in different tree fractions as described above.

The stock of exchangeable base cations and pH in the soil

The total stock of exchangeable cations in the humus layer and in the mineral soil down to 50 cm depth was calculated using data from the Swedish Forest Soil Inventory (SFSI) from 2003-2012. The calculations were made for in total 3618 plots on forestland, while plots on peatland were excluded. Data from four horizons in the soil profile were used in the calculations; humus layer, 0-10 cm (M10), 10-20 cm (M20) and 55-65 cm (M65) in the mineral soil. The entire depth of the humus layer was sampled by coring, while non-volumetric samples were obtained from the mineral soil (SLU 2012). The soil samples were dried at 40 °C and the fine fraction (<2 mm) was separated by sieving. Exchangeable base cations (Ca^{2+}, Mg^{2+}, K^+ and Na^+) were extracted with 1 M ammonium acetate at pH=7 and concentrations determined using ICP-AES spectrometry. The pH_{H2O} in the mineral soil was determined in a slurry consisting of 5 g soil and 25 ml deionised water, using a Metrohm 855 Robotic Titrosampler with an Aquatrode Plus combined pH electrode.

The bulk density of the mineral soil layers (BD_M) was calculated using a pedotransfer function (Nilsson and Lundin 2006):

$$BD_M = 2.07 * d_M + 1546.3\ e^{-0.3103\sqrt{C_M}}$$

, where d_M is the mean depth of the soil layer (cm), and C_M is the total carbon concentration (%) of the soil layer. The relative volumetric stone content was calculated using empirical functions based on the relationship between stone volumetric content determined by probing, soil type and surface boulder frequency (Stendahl et al. 2009).

The amount of charge equivalents of exchangeable base cations in the humus layer (BC_h) in $mol_c\ ha^{-1}$ were calculated with the function:

$$BC_h = \frac{m_h \sum_{i \to n}^{n=4} c_{h,i}}{a_h}$$

, where m_h is the weight of the fine fraction of the humus (h) sample (kg ha^{-1}), c_h is the equivalent concentration of exchangeable base cations (mol_c kg^{-1}), and a_h is the total sampled area (ha) (area of soil corer * number of cores sampled). The amounts of the base cations in the sampled mineral soil layers (BC_{M10}, BC_{M20}, BC_{M65}) were calculated with the function:

$$BC_M = (1 - sv)\, t_M\, BD_M\, 10^{-4} \sum_{i \to n}^{n=4} c_{M,i}$$

, where t_M is the thickness of soil layer (m), sv is the relative volumetric stone content, BD_M is the bulk density (kg m^{-3}) and c_M is the concentration of ion equivalents of the exchangeable base cations (mol_c kg^{-1}).

The exchangeable base cations stock in the section of the soil profile where no samples are taken (20-55 cm) were estimated through interpolation between the M20 sample and the M65 sample.

The proportion of acidified soils at the county level was calculated on data from SFSI where an acidified soil is defined by having a $pH_{H2O}<4.5$ in the B-horizon or a $pH_{H2O}<4.75$ in the C-horizon (Gustafsson et al. 2001).

Deposition of acidity
The acidity in deposition (mol_c ha^{-1}) was estimated from the deposition of non-sea salt SO_4^{2-} and non-seasalt Ca^{2+} according to:

$$acidity = SO_4^{2-} - Ca^{2+}$$

We use the following assumptions:

- The origin of non-sea salt SO_4^{2-} is 100% sulphuric acid (H_2SO_4) formed in the atmosphere
- Non-sea salt Ca^{2+} is derived from Ca^{2+} containing substances with a liming effect, e.g. CaO, corresponding to two hydroxide ion (OH^-) equivalents per Ca^{2+}.
- The acidification effect by N deposition is negligible due to approximately equal deposition of NH_4^+ and NO_3^- (see above).
- Other cations and Cl^- are assumed to originate from sea salt.

The relevance of using SO_4^{2-} minus Ca^{2+} as an approximation for acidity was tested with deposition data where SO_4^{2-}, Ca^{2+} and acidity were quantified. Data from EMEP (EMEP 2013) were used for deposition of sulfur (S) during the period from 1980 to 2008 and were aggregated to county level averages. EMEP data were available from the period 1980 to 2008. Therefore, we assumed that the deposition for the two periods 1955 to 1980 and 2008 to 2010 changed proportionally in the same way as the deposition at Gårdsjön estimated by Schöpp et al. (2003).

Deposition of Ca^{2+} was obtained from SMHI (SMHI 2013) that has published data for the entire country. We compiled data from 1998 for each county and assumed that the relationship between Ca^{2+} and SO_4^{2-} in deposition remained the same during the whole time

period from 1955 until 2010. The assumption of proportionality between non-seasalt SO_4^{2-} and Ca^{2+} deposition is supported by data in Hedin et al. (1994) that shows that deposition of SO_4^{2-} and base cations decreased similarly over the studied period and where Ca^{2+} was the dominating non-sea salt base cation.

Statistics

The plot based sampling design of the NFI was the basis of the estimations (Fridman et al. 2014). This sampling design was introduced in the NFI in 1953 and has remained relatively unchanged since then, with the addition of permanent plots in 1983 where the SFSI is carried out. During the entire study period 1955-2010, the inventory has covered the entire country annually with plots that are organized into sample plot clusters (i.e. tracts), utilizing a stratified sampling intensity with less intense sampling towards the north according to 5 regionalized strata. Ratio estimators with respect to area were applied in order to get unbiased estimates, accounting for sample-plot area, the sampling intensity and land use distribution of the stratum. Moving five-year averages were used for the time-series of the forest data.

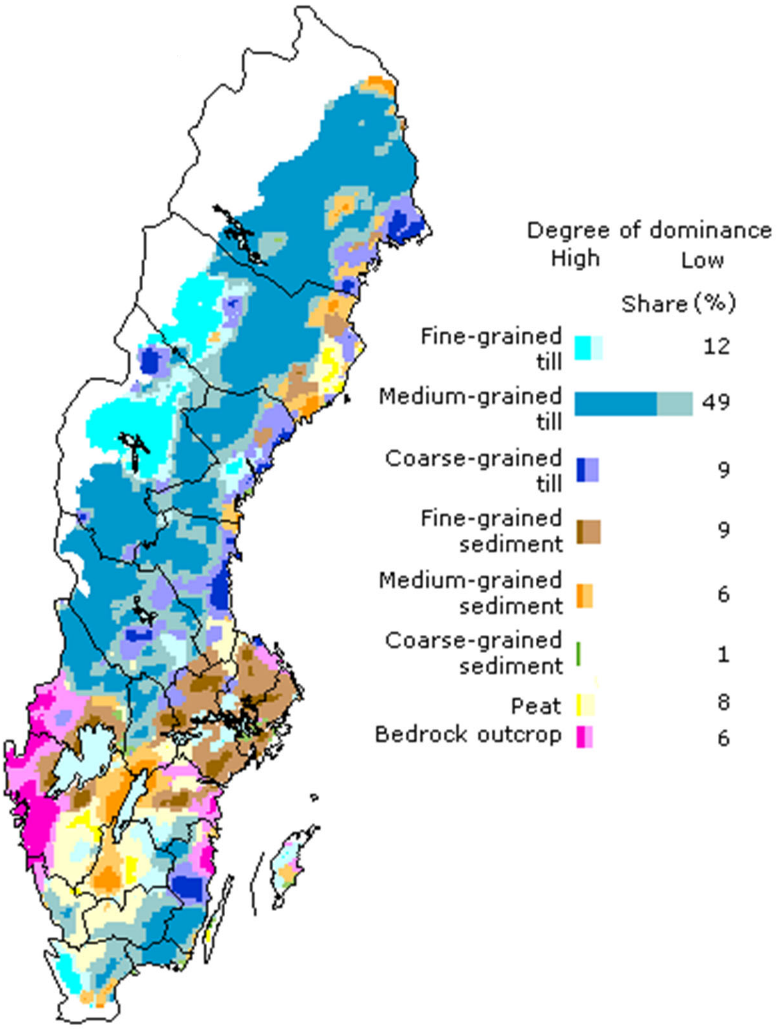

Figure S1. Dominating soil parent material texture class. Data from the Swedish Forest Soil Inventory 1983 to 1989. Representative for forest soils only.

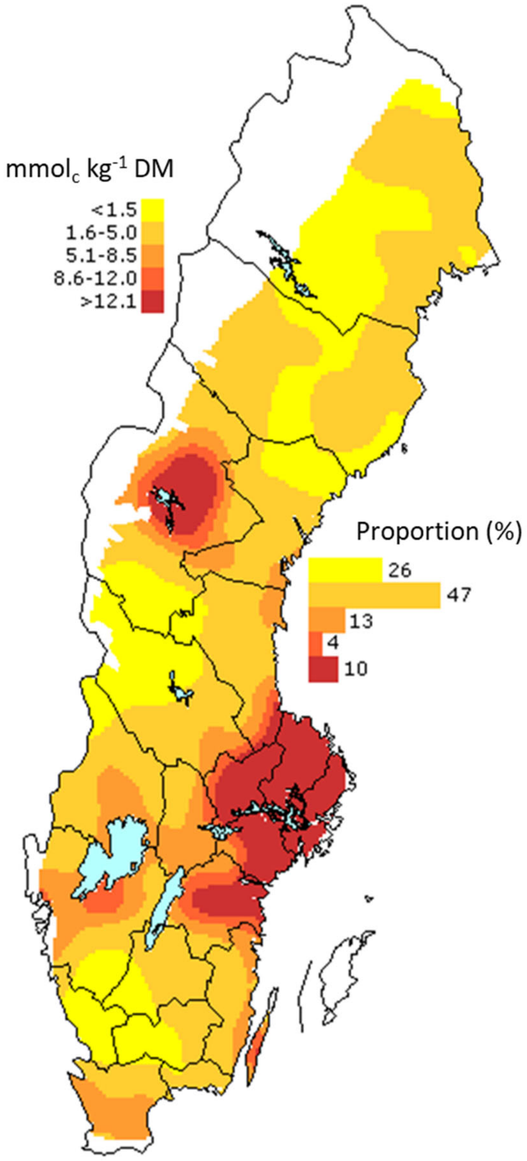

Figure S2. Exchangeable Ca in the C horizon of Swedish forest soils. Data from the Swedish Forest Soil Inventory 2003 to 2012. Representative for forest soils only.

References

Akselsson, C., and S. Belyazid. 2018. Critical biomass harvesting – Applying a new concept for Swedish forest soils. *Forest Ecology and Management* 409: 67-73.

EMEP. 2013. EBAS. http://ebas.nilu.no/. Access date: 2020-05-20.

Ferm, M., L. Granat, M. Engardt, G. Pihl Karlsson, H. Danielsson, P. E. Karlsson, and K. Hansen. 2019. Wet deposition of ammonium, nitrate and non-sea-salt sulphate in Sweden 1955 through 2017. *Atmospheric Environment: X* 2: 100015.

Fridman, J., S. Holm, M. Nilsson, P. Nilsson, A. Ringvall, and G. Ståhl. 2014. Adapting National Forest Inventories to changing requirements – the case of the Swedish National Forest Inventory at the turn of the 20th century. *Silva Fennica* 48: 1-29.

Futter, M. N., E. Ring, L. Högbom, S. Entenmann, and K. H. Bishop. 2010. Consequences of nitrate leaching following stem-only harvesting of Swedish forests are dependent on spatial scale. *Environ Pollut* 158: 3552-3559.

Gustafsson, J. P., E. Karltun, U. Lundström, and O. Westling. 2001. Urvalskriterier för bedömning av markförsurning (Criterias for assessing soil acidification). Rapport 11D, Jönköping, Sweden. https://cdn.abicart.com/shop/9098/art39/4646039-dfd223-1689.pdf.

Hedin, L. O., L. Granat, G. E. Likens, T. Adri Buishand, J. N. Galloway, T. J. Butler, and H. Rodhe. 1994. Steep declines in atmospheric base cations in regions of Europe and North America. *Nature* 367: 351-354.

Högberg, P., T. Näsholm, O. Franklin, and M. N. Högberg. 2017. Tamm Review: On the nature of the nitrogen limitation to plant growth in Fennoscandian boreal forests. *Forest Ecology and Management* 403: 161-185.

Iwald, J., S. Löfgren, J. Stendahl, and E. Karltun. 2013. Acidifying effect of removal of tree stumps and logging residues as compared to atmospheric deposition. *Forest Ecology and Management* 290: 49-58.

Löfgren, S., U. Grandin, and S. Stendera. 2014. Long-term effects on nitrogen and benthic fauna of extreme weather events: Examples from two Swedish headwater streams. *Ambio* 43: 58-76.

Marklund, L. G. 1988. Biomassafunktioner för tall, gran och björk i Sverige (Biomass functions for pine, spruce and birch in Sweden). Report 45, Department of Forest Survey, Swedish University of Agricultural Sciences, Umeå.

Nilsson, T., and L. Lundin. 2006. Uppskattning av volymvikten i svenska skogsjordar från halten organiskt kol och markdjup (Estimations of bulk density from soil organic carbon concentrations and soil depth in Swedish forest soils). Rapporter i skogsekologi och skoglig marklära 91, Swedish University of Agricultural Sciences, Uppsala, Sweden.

Petersson, H., and G. Ståhl. 2006. Functions for below-ground biomass of Pinus sylvestris, Picea abies, Betula pendula and Betula pubescens in Sweden. *Scandinavian Journal of Forest Research* 21: 84-93.

Schöpp, W., M. Posch, S. Mylona, and M. Johansson. 2003. Long-term development of acid deposition (1880–2030) in sensitive freshwater regions in Europe. *Hydrol. Earth Syst. Sci.* 7: 436-446.

SLU. 2012. Fältinstruktion 2012 (Field manual 2012). Swedish University of Agricultural Sciences, Umeå and Uppsala, Sweden.

SLU. 2017. Skogsdata 2017 (Forestry statistics 2017). Swedish University of Agricultural Sciences/Official Statistics of Sweden, Umeå, Sweden. https://www.slu.se/globalassets/ew/org/centrb/rt/dokument/skogsdata/skogsdata_2017.pdf.

SMHI. 2013. Luftmiljö - Halter och deposition - Modelldata (Air quality - Concentrations and deposition - Model data). https://www.smhi.se/data/utforskaren-oppna-data/luftmiljo-halter-och-deposition-modelldata. Access date: 2020-05-05.

Stendahl, J., L. Lundin, and T. Nilsson. 2009. The stone and boulder content of Swedish forest soils. *Catena* 77: 285-291.

Swedish Forest Agency. 2014. Skogsstatistisk årsbok 2014 (Swedish Statistical Yearbook of Forestry 2014). Swedish Forest Agency/Official Statistics of Sweden, Jönköping, Sweden.
